# Acknowledgment to Reviewers of *Sports* in 2021

**DOI:** 10.3390/sports10020020

**Published:** 2022-01-31

**Authors:** Sports Editorial Office

**Affiliations:** MDPI AG, St. Alban-Anlage 66, 4052 Basel, Switzerland

Rigorous peer-reviews are the basis of high-quality academic publishing. Thanks to the great efforts of our reviewers, *Sports* was able to maintain its standards for the high quality of its published papers. Thanks to the contribution of our reviewers, in 2021, the median time to first decision was 22 days and the median time to publication was 46 days. The editors would like to extend their gratitude and recognition to the following reviewers for their precious time and dedication, regardless of whether the papers they reviewed were finally published:
Aaron ScanlanJosé Ramón Alvero-Cruz Alvero CruzAbdul Rashid AzizJosé V. Gutiérrez-ManzanedoAdam GormanJose Vilaça-AlvesAdam WhiteJoshua P. BaileyAdela BadauJozef SimenkoAdrian A. ValverdeJuan De Dios Benítez SilleroAgnieszka Zembrón-ŁacnyJuan José Gonzalez GerezAïmen KhacharemJuan Mielgo-AyusoAjay PalaganiJulia BoneAlban Fouasson-ChaillouxJulio CallejaAlejandro Martínez-RodriguezJulio CostaAlejandro Pérez-CastillaJurdan MendiguchiaAlejandro Sánchez-PayJustus HaucapAleksandra KrólikowskaKatie HeinrichAleksandra RogowskaKatsunori NakamuraAlessandro FeolaKazuhiro P. IzawaAlex Buoite StellaKazuki FukuiAlfonso Penichet-TomásKevin TillAli BoolaniKhaled TrabelsiAlina KuryłowiczKonstantinos PapanikolaouAlvaro Lopez-SamanesKristina PennistonAnastassios PhilippouKrzysztof JamroziakAndras PappKrzysztof KusyAndré NovoKrzysztof Sas-NowosielskiAndrea FuscoKuen-Chang HsiehAndrea VitaliLachlan MitchellAndrew D′LugosLarisa-Loredana DragoleaAndrew HatchettLaurent MetzingerAndrew HultonLawrence JudgeAndrew J. HarrisonLeonor GallardoAndrew LavenderLiliana Szyszka-SommerfeldAndrzej MastalerzLiudmila I. Gerasimova-MeigalAnna LeopoldLuca ArdigòAnna Tabęcka-ŁonczyńskaLuca CavaggioniAnthony HesselLuca Paolo ArdigòAntonio CicchellaLuciana LabancaAntónio MendoLuigi MeccarielloArgyris ToubekisLuis CarrascoArkadiusz StanulaLuis Javier Chirosa RíosArkaitz CastañedaLuís RamaArne GüllichŁukasz RadzimińskiArthur ChavesMagdalena GórnickaAshley ArteseMałgorzata BronikowskaAsrat AmnieMalgorzata SyczewskaAthos TrecrociManuel Albornoz-CabelloBadicu GeorgianMarc ArgilésBasilio PueoMarc LochbaumBlanca De-la-Cruz-TorresMarco GervasiBrach PostonMarco J.M. HoozemansBrandon J. SawyerMarcos Mecías-CalvoBritton W. BrewerMargaret JonesBruce W. CraigMaria Francesca PiacentiniBruno TravassosMaría Luisa Zagalaz SánchezBryanne N. BellovaryMaría M. Morales Suárez-VarelaBud CooperMarieke WoutersCaio Victor SousaMário Jorge CostaCarl SingletonMark Elisabeth Theodorus WillemsCarlos David Gómez-CarmonaMark RussellCarlos Eduardo Barro GonçalvesMarko StupinCarlos López-de-CelisMartin OtisCarlos Romero MoralesMasatoshi NakamuraCarole ComettiMathieu JozwiakCesar I. Fernández LázaroMati PääsukeChe FornusekMatt R. CrossChia-Hua KuoMatteo Levi MicheliChing-Ting HsuMatthew CookChong ChenMatthew H. ZimmermanChristian RoquesMatthew J. ReevesChristoph TriskaMatthew RogatzkiChristopher BallmannMeike StrekerChristopher KirkMicah GrossChristos PaizisMichael J. DuncanClaudia LeongMichael KeinerClayton L. CamicMichael KingsleyCornelis Johannes De RuiterMichael OlsonCourtney E. ShellMichaela ŠiklováCraig A. HorswillMichał BronikowskiCristina CortisMichał KrzysztofikCristina Petisco-RodríguezMichal StarczewskiDagmar PavluMichale KoehleDaichi SumiMichalina BłażkiewiczDaichi YamashitaMichel MarinaDale ChapmanMichele BoffanoDamien YoungMiguel Angel Gómez-RuanoDaniel A. BaroneMike ClimsteinDaniel BecqueMikel Pérez GutiérrezDaniel HackettMikolaj DabrowskiDaniel López-LópezMilivoj J. DopsajDariusz BoguszewskiMitchell LachlanDariusz NowakMojtaba KavianiDavid BehmMotomu NakashimaDavid BerryNai-Jen ChangDavid H. FukudaNatasa ZenicDavid NiederseerNavarro Gómez NoeliaDavid ZweikerNicholas GistDeldicque LouiseNicolas BabaultDennis Peter BornNikolai Baastrup NordsborgDiego Alonso-FernandezOzcan EsenDiego Fernández-LázaroPablo BurilloDolores D. GuestPål LagestadDonatella Di CorradoPanagiotis TsimeasDorota Kostrzewa-NowakPantelis T. NikolaidisDorota LewczukPaolo Riccardo BrustioDuarte Henriques-NetoPasqualino Maietta LatessaDustin Russel SlivkaPaul DorianEleftherios KellisPaulo Vicente JoãoElena Escolano-PérezPaweł ChmuraEmanuela Gualdi-RussoPawel MacekEmiliano CèPedro FonsecaEmmanuel Navarro-FloresPedro L. ValenzuelaEnrique RochePedro Sáenz-LopezEnza SperanzaPedro Valdivia-MoralEric BerksonPei-Lun KuoEric MosierPeter HofmannEric SobolewskiPeter SarcevicErica MenegattiPetr StastnyErik LindPetros BotonisErika ZemkováPhil ChilibeckEuphrosyni KoutsourakiPhil J. HandcockFabiola SpolaorPierpaolo IodiceFelice SiricoPietro PicernoFernando González-MohínoPiotr GawdaFilipe Manuel ClementePitre C. BourdonFotini ArabatziPiyush SharmaFrancesca LatinoPrasenjit SahaFrancesco AgostiniRafael BurgueñoFrancesco FischettiRafael OliveiraFrançois LalondeRamos-Petersen LauraFrank E. NelsonRaquel Leirós-RodríguezFrank WyattRaul DomínguezFrantišek LopotRaúl Tárraga MínguezFraser CarsonRebecca WilliamsGaetana NapolitanoRen-Jay SheiGarry KuanRicardo Manuel Pires FerrazGary BrickleyRobert Alan SloanGary LiguoriRobert F. BentleyGennady KolesnikovRoland Van Den TillaarGeoffrey IstasRomán NuvialaGerard McMahonRomualdas MalinauskasGerd SchmitzRosario BaroneGiampaolo SantiRussell BestGiancarlo CondelloRyan ColquhounGianluca EspositoRyan M. SappGianpiero GrecoRyan WornGiovanna GhianiRyu NagaharaGiuseppe CaminitiSabrina Donati ZeppaGiuseppe CoratellaSai-fu FungGoran KuvačićSalvador Romero-ArenasGovindasamy BalasekaranSamo RauterGregory BogdanisSamuel MettlerGregory J. GrosickiScot RaabGustavo Vieira De OliveiraScott C. ForbesGusztáv FeketeScott MazzettiHaneul LeeScott W. TalpeyHelder Miguel FernandesSébastien MurerHenk KoppelaarSergio López-GarcíaHenri TilgaSergio Selles-PerezHenrique NascimentoSeungyong LeeHenry LukaskiShaher A. I. ShalfawiHermann ToplakShalaka MulherkarHugo SarmentoShih-yu LeeIbrahim HassanShinsuke NirengiIgnacio RefoyoShin-Tsu ChangIgor JukićSihui MaIoana MozosSilvio LorenzettiIrene MargaritisSime VersicIrene Torres-SánchezSoo Jin YangIsmael GuardadoStefano AmatoriIván Chulvi-MedranoStephan Van Der ZwaardJacek JurkojćStéphane PerreyJack CannonStephen CornishJackson FyfeStephen IvesJacob M. WilsonSteve ThompsonJae-Sung YooSzabo AttilaJakub KortasTadashi ItoJakub S. GąsiorTadeusz AmbrozyJames M. NobleTarak DrissJan MieszkowskiTatiana GörigJanet ZhangTatsuro EgawaJarosław CholewaTeresa ZwierkoJason LakeThanutchaporn KumrungseeJasper VerheulThemistoklis TsatalasJavier Sevil-SerranoThomas BiasJeffrey S. MartinThórarinn SveinssonJerry MayhewTim MarjoribanksJesus Prieto-LloretTimothy B. DaviesJesus Vicente GiménezTimothy J. SuchomelJisu KimTobias DünnwaldJo KatoTomás FreitasJoão PetricaTomás UrbánJohn Andree BabrajTomasz GabryśJohn F. T. FernandesTomasz KlekielJohn J. GuersTomasz WolnyJohn Luke PryorToshinobu TakedaJohneric W. SmithToshio MoritaniJohnny PaduloTsering StobdanJon OliverVaclav BuncJongsang SonWeisheng ChiuJoohyung LeeWesley KephartJose A. BragadaWojciech J. CynarskiJosé Antonio LoyaXiao ChangJosé BonalXiaohu HuangJosé Carlos Fernández-GarcíaXurxo DopicoJosé Carmelo AdsuarYong-Seok JeeJose Ignacio Priego QuesadaYoshinari UehaJosé Ramón Alvero Cruz


